# Dedifferentiated liposarcoma of the orbit

**DOI:** 10.1016/j.ajoc.2023.101980

**Published:** 2023-12-26

**Authors:** Angela J. Oh, Robert A. Goldberg, Ben J. Glasgow

**Affiliations:** a Departments of Ophthalmology, Jules Stein Eye Institute, University of California, Los Angeles, CA, USA; b Pathology & Laboratory Medicine, Jules Stein Eye Institute, University of California, Los Angeles, CA, USA

**Keywords:** Liposarcoma, Dedifferentiated liposarcoma, Well-differentiated liposarcoma, Adipocytes

## Abstract

**Purpose:**

To present a rare case of dedifferentiated liposarcoma of the orbit.

**Observations:**

A 61-year-old male complained of left-sided proptosis, diplopia, and limited ocular motility for two years. Biopsy results at that time were suggestive of an atypical lipomatous neoplasm. Ten years later, he presented with increase in size of the mass and worsening of his symptoms. Imaging showed a multi-lobulated mass in the left orbit involving the intraconal, medial, and anterior orbit. Decompression and orbitotomy with biopsy were performed to debulk the mass. Pathology showed a low-grade well-differentiated liposarcoma and the patient was monitored thereafter annually. Eight years later, he complained of persistent proptosis and mass effect from the tumor resulting in ptosis and diplopia and underwent orbital exenteration. Histopathological analysis of the exenterated orbit revealed a focal area of dedifferentiated liposarcoma.

**Conclusions and importance:**

Dedifferentiation of an orbital mass can occur as a late complication years after the diagnosis of well-differentiated liposarcoma. Compared to the previously published cases of orbital liposarcoma, this presentation shows a prolonged timeline prior to dedifferentiation (18 years after initial diagnosis). Symptoms of growth or invasive features could indicate dedifferentiation and should warrant a biopsy.

## 1 Introduction

Primary orbital liposarcoma is a rare soft tissue malignancy that can present in five subtypes: myxoid, well-differentiated, pleomorphic, round cell, and dedifferentiated. Dedifferentiated liposarcoma (DDLS) is a rare subtype and compared to the well-differentiated type, is associated with worse prognosis and increased rates of recurrence.^1^ DDLS typically occurs in the retroperitoneum or extremities. Cases in the orbit are extremely rare.^2^, ^3^, ^4^, ^5^, ^6^, ^7^, ^8^ Here we report a case of dedifferentiation of a primary orbital well-differentiated liposarcoma eighteen years after initial diagnosis.

## 2 Case report

A 61-year-old male reported a two-year history of a progressively growing mass of the left orbit with associated diplopia. He denied other vision changes or pain and had no known medical problems. Biopsy results from an outside hospital showed lipoblasts and bone infiltrated by atypical cells, consistent with an atypical lipomatous neoplasm.

**Fig. 1:**
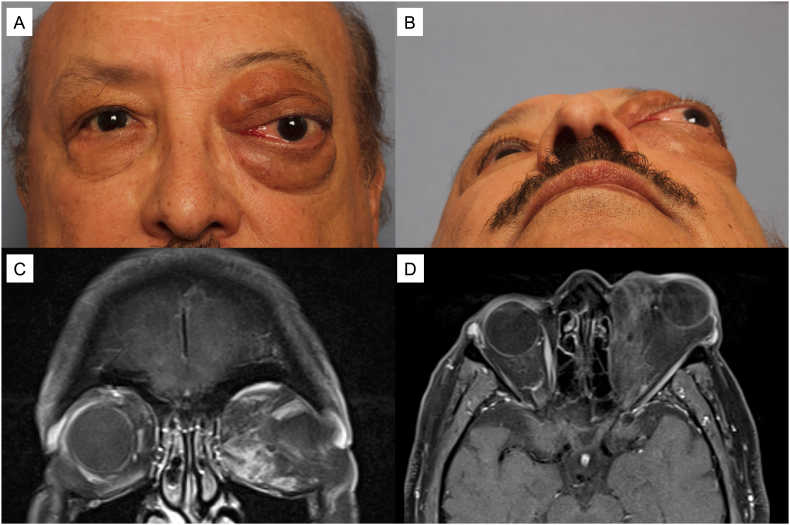
External images and MRI images of a 61-year-old patient with primary dedifferentiated liposarcoma of the left orbit. (A) There is significant left eye proptosis with surrounding edema of the upper and lower lid due to an irregularly shaped mass in the orbit. The photograph displays the patient examined in primary gaze. (B) Worm's eye view emphasizes the degree of proptosis and asymmetry. (C) T2-weighted magnetic resonance imaging of the orbit (coronal view) post contrast with fat suppression shows an irregular intraconal mass with variable areas of contrast enhancement. (D) Axial view.

Ten years later, he presented to us with worsening left-sided proptosis and discomfort from the orbital mass. On examination, best corrected visual acuity was 20/25 in the right eye and 20/20 in the left eye. Pupils were equal, round, and reactive with no relative afferent pupillary defect and confrontational visual field testing was full bilaterally. Motility exam showed restriction of the left eye in all gazes, worse with upgaze and adduction. External exam showed a soft, palpable medial mass in the left orbit with prominent herniated orbital fat (Fig. 1A). There was proptosis (Hertel base 101mm, 16mm right eye and 26mm left eye) and 2mm of inferior scleral show in the left eye (Fig. 1B). Slit lamp exam showed medial conjunctival injection with chemosis of the left eye. There was no asymmetry in sensation of the face and no pulsating or vascular features of the mass.

Magnetic resonance imaging (MRI) of the orbit showed a multi-lobulated orbital tumor with worm-like configuration in the deep orbit involving the intraconal, medial, and anterior orbit (Fig. 1C and D). T1-weighted imaging showed heterogeneous contrast enhancement of the lesion with iso-intense and hyperintense segments. There was no evidence of bony erosion of the orbital walls. The patient's long-standing history (10+ years), lack of infiltrative features, and imaging suggested a relatively slow growing process. However, given the patient's progression of symptoms, a left orbital decompression and orbitotomy were performed for debulking. Pathology showed multiple tan-yellow nodular irregular soft tissue segments measuring 5.3 × 5.0 × 0.9 cm in aggregate. There were atypical adipocytes surrounding dense fibrosis consistent with low-grade well-differentiated liposarcoma. Radiation oncology did not recommend adjunctive therapy at this time. The risk of recurrence was discussed with the patient, and he was monitored closely with orbital imaging.

Five years later, the patient noticed enlargement of the lesion. Visual acuity was decreased to 20/30 in the left eye. Hertel measurements (base 110mm) were 22mm in the right eye and greater than 35 mm in the left eye. Repeat imaging did not show significant change in size of the lesion. T1-weighted imaging showed variable contrast enhancement similar to prior scans. Given his symptoms were not severe and proptosis and fullness were not dramatically worse, further surgical debulking was deferred. Two years later, the patient complained of new left-sided ptosis. Margin to reflex distance 1 was 3 mm on the right eye and 0 mm on the left. He had normal levator function to 14mm bilaterally with mild lagophthalmos medially in the left eye. The patient underwent anterior ptosis repair with a permanent medial tarsorrhaphy.

One year later, the patient returned with increased proptosis and recurring left-sided ptosis. He also had intermittent binocular diplopia and left-sided temple pain. Visual acuity decreased to 20/50 in the left eye. There was persistent proptosis with recurrent ptosis of the left eye (margin to reflex distance 1 of the left eye was −2 mm). The patient complained of impairment of his activities of daily living due to his diplopia and was bothered by the appearance of the left orbit. Revision ptosis surgery was offered but declined given the possibility that this could worsen his diplopia. Based on the unlikely efficacy of medical treatment or radiation and the lack of any good globe-sparing surgical option, decision was made with the patient to perform orbital exenteration for pain control and possible cure.

**Fig. 2:**
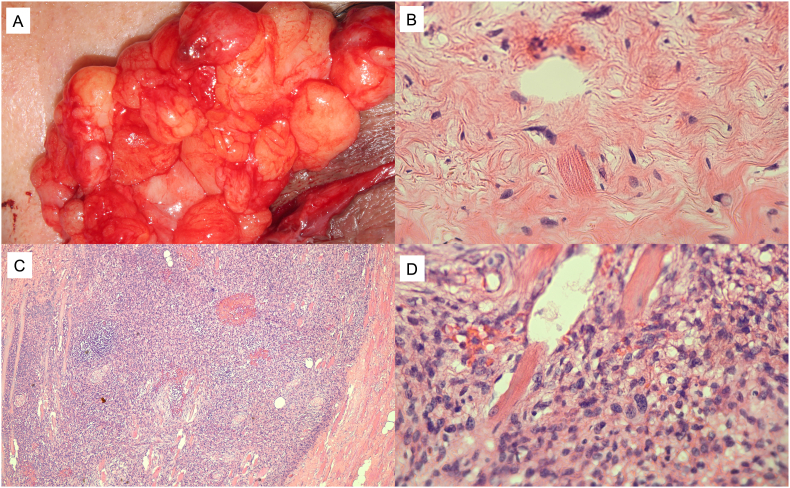
Pathology of dedifferentiated liposarcoma of the orbit. (A) Gross pathology photos intraoperatively during initial debulking surgery shows the lipogenic component of the tumor. (B) Hematoxylin-eosin stain features atypical adipocytes infiltrating striated muscle and fibrous tissue consistent with well-differentiated liposarcoma. (C) Focal area of dedifferentiated liposarcoma following orbital exenteration. (D) High magnification shows pleomorphic spindle-shaped cells with mitotic figures.

Intraoperatively, the lipogenic mass measured 6.6 × 3.2 × 2.2 cm (Fig. 2A). Pathology showed atypical fibroadipose tissue with spindle shaped cells with a focal area of dedifferentiated tumor (largest dimension of 6 mm) centrally distant from all margins (Fig. 2B and C). Surrounding mitotic figures confirmed the diagnosis of dedifferentiated liposarcoma (Fig. 2D). Anti-pankeratin staining was negative. Fluorescence in situ hybridization analysis (FISH) results were inconclusive and MDM2 showed no analyzable results. Two months following exenteration, the patient reported no pain and improved symptoms. Follow up will continue with annual orbital imaging.

## 3 Discussion

Primary orbital DDLS is an extremely rare diagnosis with only seven reported cases found in the literature (Table 1).^2^, ^3^, ^4^, ^5^, ^6^, ^7^, ^8^ In most of these cases, diagnosis was made on initial biopsy. Thus, a preceding low-grade or well-differentiated liposarcoma (WDLS) has not been well documented. Our case provides a timeline of dedifferentiation of a primary orbital liposarcoma eighteen years after initial diagnosis.

**Table 1:** Reported cases of primary dedifferentiated liposarcoma (DDLS) of the orbit.

Author	Age	M/F	Signs	Initial histopathology	Time from initial biopsy to diagnosis	XRT	OE	Recur
Cai	54	F	Proptosis, amblyopia	WDLS with focal spindle cell element *History of ossifying fibroma 4 years prior with recurrence after 3 years	4 years	No	Yes	None
Stiglmayer	55	F	Proptosis	DDLS	Immediate	Yes	Yes	None
Saeed	56	F	Proptosis, diplopia	DDLS	Immediate	Yes	Yes	None
Zhang	23	F	Proptosis, ophthalmoplegia	DDLS	Immediate	Yes	No	None
Andrea	46	M	Upper lid edema	DDLS	Immediate	Yes	Yes	None
Peng	56	F	Proptosis	Lipoma	4 years	No	No	None
Yamazaki	41	M	Proptosis, ophthalmoplegia,	DDLS	Immediate	No	No	None
Oh	61	M	Proptosis	WDLS	18 years	No	Yes	None

F: Female; M: male; WDLS: well-differentiated liposarcoma; DDLS: Dedifferentiated liposarcoma. XRT: radiation; OE: orbital exenteration.

Typically, patients with orbital DDLS present with proptosis (Table 1). Diplopia and limitation of eye movements, such as in our case, are less common but can occur.^4^^,^^5^^,^^8^ Imaging can be helpful for diagnosis as well as to monitor change over time. On MRI, DDLS presents with coexistent areas of lipomatous and non-fatty solid components. Increased contrast enhancement may be a clue to distinguish DDLS from WDLS, although this is not specific.^7^ Initial T-1 weighted imaging of our patient showed non-homogenous areas of hyperintensity. Contrast enhancement appeared variable on subsequent imaging. No distinct nodule was identified. Although this was not done in our patient, positron emission tomography can be used to assess the level of fluorodeoxyglucose uptake, which is increased in DDLS compared to WDLS.^9^ Orbital imaging might include liposarcoma in the differential diagnosis of an orbital mass but histopathology is needed for confirmation. DDLS contains areas of undifferentiated pleomorphic or spindle-shaped cells with mitotic figures.^1^ In our case, the coexistence of well-differentiated areas surrounding the area of crowded poorly differentiated pleomorphic or spindle cells is characteristic. Diagnosis can be confirmed with immunohistochemistry and FISH evaluation of MDM2.

Management of DDLS in the orbit is challenging given its rarity. Compared to the more common WDLS, DDLS has higher recurrence rates and worse prognosis.^2^ In WDLS, 30 % of patients experience local recurrence with partial excision and only 5 % metastasize.^2^ In contrast, DDLS has a high recurrence rate of 41 % and a disease specific mortality of 28 % for tumors of the extremities.^7^ Fortunately, none of the reported cases of primary orbital DDLS showed local recurrence or metastasis (Table 1). Even still, four of the seven patients underwent orbital exenteration. This is done likely to achieve complete tumor control and prevent residual growth.^1^ Our patient also underwent an exenteration, although the reason for this was more for symptomatic tumor growth. In contrast, patients with WDLS are able to pursue globe-sparing therapies given the lower rates of metastasis. Four of the seven orbital DDLS cases completed radiotherapy but this may not improve survival or rates of metastasis (Table 1).^7^ In our case, neither radiation nor chemotherapy was recommended. Response to chemotherapy is limited and none of the cases of primary orbital DDLS underwent chemotherapy.^1^

While the majority of DDLS occurs de novo, 10 % arise from dedifferentiation of WDLS.^1^ One case series found 14 % (22 of 155 cases) of DDLS developed in the setting of pre-existing WDLS.^10^ Dedifferentiation can occur at any site but the risk varies depending on the location of the initial tumor. The most common location is the retroperitoneum with a risk of up to 28 %. Compared to DDLS of the extremities, tumors of the retroperitoneum also have worse prognosis. Rarely, dedifferentiation is seen in the superficial soft tissue and the head and neck region.

Furthermore, the risk of dedifferentiation may be time-dependent. It appears to take years for dedifferentiation to develop and/or present itself. One study found an average latent period of 7.7 years (range 1–23 years, median of 6 years) after the diagnosis of a preexisting WDLS.^10^ Another series of dedifferentiated sarcomas in the retroperitoneum, thigh, or groin found an average interval period between diagnosis and dedifferentiation of 11 years (range 2–18 years, median of 9 years).^11^ In our patient, dedifferentiation was discovered eighteen years after diagnosis of WDLS, which falls within the upper limit range of latent period of both case series. Dedifferentiation may also be more likely with prolonged chronic disease. This makes sense particularly in the retroperitoneum, where tumors are larger, less likely to be completely excised, and can have indolent growth for years before becoming symptomatic. The orbit is unique because symptoms may be apparent early on. However, complete excision is challenging without sacrificing the globe. Our patient lived with this orbital mass for over 20 years, supporting the theory of dedifferentiation after many years. Diagnosis of DDLS was made only after exenteration. Our case highlights the possibility of an extended time course before dedifferentiation of a primary orbital WDLS. We found one report of dedifferentiation in the orbit and the brain from a metastatic lesion from a primary liposarcoma of the thigh resected six years prior.^12^ Of the seven reported cases of primary orbital DDLS, all but two presented as DDLS on initial pathology (Table 1). In these 2 cases the timelines are imprecise because of prior lesions that were not WDLS.^2^^,^^6^ In our patient, there was a clear interval of time (18 years) between WDLS and the development of DDLS. One may argue that our initial diagnosis was incomplete given we did not excise the whole tumor. However, pathology from the exenteration showed a focus of dedifferentiation on step sections, suggesting recent transformation rather than a remote history.

Dedifferentiation can occur in primary orbit liposarcomas after 18 years, emphasizing the importance of long-term follow up. In our patient, diplopia, proptosis, and tumor growth were driving factors in the decision to pursue exenteration, which led to the discovery of DDLS. His initial biopsy did not show areas concerning or suggestive of DDLS on histology. On follow up exams, he did not have an acute change in his symptoms but rather a slow progressive worsening that prompted exenteration. It is possible that the first sign of dedifferentiation was when the patient presented with worsening proptosis five years after his biopsy with us, but we did not suspect dedifferentiation at this time. There was no evident growth of the lesion on imaging and the patient's symptoms were not severe enough to pursue repeat debulking. Given the possibility of early dedifferentiation of orbital WDLS, one might consider a low threshold for repeat debulking. Progression of symptoms may be the first sign of dedifferentiation and should prompt consideration for repeat biopsy.

## 4 Conclusions

Primary orbital DDLS is rare and can arise from WDLS many years after initial diagnosis. Significant growth or invasive features should engender consideration for repeat biopsy or debulking to assess for dedifferentiation.

## Patient consent

Informed consent for publication was obtained.

## Declaration of competing interest

The authors declare that they have no known competing financial interests or personal relationships that could have appeared to influence the work reported in this paper.
